# Boosting the velocity detection limit of 3D single‐cell tracking time‐lapse MRI by balanced SSFP imaging

**DOI:** 10.1002/mrm.30553

**Published:** 2025-05-23

**Authors:** Enrica Wilken, Asli Havlas, Lydia Wachsmuth, Max Masthoff, Clemens Diwoky, Cornelius Faber

**Affiliations:** ^1^ Clinic of Radiology University of Münster Münster Germany; ^2^ Institute of Molecular Biosciences University of Graz Graz Austria

**Keywords:** bSSFP, compressed sensing, high temporal resolution, radial sampling, single‐cell tracking, time‐lapse MRI

## Abstract

**Purpose:**

Time‐lapse MRI allows for the dynamic tracking of single iron‐labeled cells. However, the time required for spatial encoding creates a temporal blur and, therefore, a limited ability to resolve moving cells. To study fast moving cells, such as rolling immune cells along the endothelium during inflammatory processes, advanced accelerated acquisition techniques are required.

**Methods:**

Balanced SSFP (bSSFP) imaging is applied to phantom and in vivo murine brain time‐lapse MRI measurements at 9.4 T. Its detection capability of moving iron‐labeled cells is compared with conventional gradient echo imaging (GRE) for 2D Cartesian sampling and evaluated for fully sampled and accelerated reconstructions with compressed sensing for 3D interleaved radial sampling in bSSFP.

**Results:**

Both phantom and in vivo time‐lapse MRI measurements show that single cells can be followed dynamically using bSSFP. High temporal resolution of less than 2 min reduces geometric distortion. The velocity detection limit increases to 0.8 mm/min in vitro and previously hidden fast‐moving cells are recovered. Interleaved 3D radial sampling enables 3D cell tracking and simultaneous imaging at varying acceleration factors. Fivefold acceleration with compressed sensing optimizes cell visibility, image quality, and temporal resolution.

**Conclusion:**

bSSFP time‐lapse MRI improves single‐cell tracking by enhancing temporal resolution. In vitro, the velocity detection limit is increased fourfold compared to conventional GRE. Interleaved 3D radial bSSFP offers whole‐brain coverage at isotropic spatial resolution and retrospective reconstruction of both fully sampled and high temporal resolution images.

## 1 INTRODUCTION

Reliable tools for single‐cell tracking are needed given the growing interest in the dynamic role of immune cells in inflammation and cancer and the development of new cell‐based therapies. Several imaging modalities, such as MR‐, optical‐, ultrasound‐ and radioisotope‐based techniques, are available to track cell movement in vivo. However, each has its disadvantages, including invasiveness, low spatial or temporal resolution, limited penetration depth, or low sensitivity.^1^, ^2^ Tracking single cells moving rapidly within blood vessels is currently only possible with intravital confocal microscopy using cranial or skinfold windows, an invasive procedure with limited FOV.^1^ Here, different motion patterns of immune cells have been observed: passive bloodstream travel of cells at velocities >12 mm/min,^3^ and active leukocyte migration along the endothelium.^4^ Monocytes begin rolling at ˜2.4 mm/min during inflammation, and non‐classical monocytes intermittently exhibit slow movement at ˜12 μm/min under healthy conditions.^4^ This “patrolling” occurs for varying durations and can be confined to areas <40 μm or span greater distances >100 μm.^4^

MRI provides a non‐invasive imaging alternative with unlimited tissue penetration, whole‐organ coverage, high spatial resolution, simultaneous anatomical information, and high soft tissue contrast.^5^, ^6^ To achieve sufficient contrast and sensitivity at the cellular level, superparamagnetic iron‐oxide nanoparticles (ION) can be used as a contrast agent to label cells.^2^, ^7^, ^8^, ^9^, ^10^, ^11^ Because of the induced magnetic field perturbations, single cells appear as signal voids in $T_{2}^{*}$‐weighted images enabling the detection of static single cells both in vitro and in vivo.^12^, ^13^, ^14^, ^15^ Several dedicated pulse sequences have been developed to visualize iron‐labeled cells,^7^ including positive contrast methods.^16^, ^17^ However, none of these methods were able to visualize the displacement of single cells within one imaging session. This was only achieved with the introduction of time‐lapse MRI.^11^, ^18^, ^19^ Through repetitive imaging, time‐lapse MRI enables dynamic in vivo tracking of single cells within the vascular system, revealing motion patterns of patrolling immune cells of different time scales and distances.^11^, ^18^, ^19^ Yet, when cells move too quickly, temporal blurring occurs, and cells moving faster than a few tens of μm/min cannot be distinguished from the background.^19^, ^20^ Although changes in the motion behavior of patrolling monocytes during an immune response can be studied,^8^, ^18^ rolling or flowing monocytes cannot yet be resolved. Recently, it has been shown that undersampling (US) k‐space, therefore, acquiring fewer data in shorter scan times, in combination with compressed sensing (CS)^21^ improves time‐lapse MRI for Cartesian and radial sampling.^20^, ^22^ Reduced frame durations of as short as 1 to 2 min increased the maximum detectable velocity of cells twofold to threefold compared to the established fully sampled (FS) Cartesian gradient echo (GRE) time‐lapse MRI sequence.^20^ Nevertheless, further acceleration of data acquisition is necessary to uncover faster moving cells and to deepen our understanding of inflammatory processes through single‐cell tracking with time‐lapse MRI.

In this work, balanced SSFP (bSSFP) imaging^23^ was applied for dynamic single‐cell tracking by time‐lapse MRI. Previous time‐lapse MRI studies have used multislice 2D GRE acquisition, enabling long TR to allow for relaxation of the background signal to create sufficient contrast with iron‐labeled cells. Here, we explore the feasibility of bSSFP imaging for time‐lapse MRI, which is superior in terms of (temporal) SNR and contrast‐to‐noise‐ratio (CNR) compared to other pulse sequences conventionally used for iron detection^24^, ^25^ and, most importantly, may enable 3D acquisition. Although contrast and performance of both GRE and bSSFP for static objects are well known, the effects of small objects moving during acquisition of one set of k‐space data are not. Therefore, first, a 2D Cartesian bSSFP sequence was used to increase temporal resolution compared to established GRE time‐lapse MRI sequences.^18^, ^19^ Second, the method was extended using a 3D radial double‐echo bSSFP sequence.^26^ Here, an interleaved acquisition scheme, with sequentially acquired subsets of all spokes, each covering k‐space uniformly, permitted retrospective reconstruction of both FS and US images. This irregular sampling further allowed additional CS reconstruction of highly accelerated images without a loss in image quality. Both sequences, 2D Cartesian and 3D radial, yielded an increase in the velocity detection limit in vitro, which was measured based on the change in signal loss single iron particles generate in phantom measurements using a rotating phantom system. In vivo time‐lapse MRI imaging the mouse brain after in vivo labeling of cells confirmed feasibility of both approaches.

## 2 METHODS

### 2.1 Phantom experiments

Cylindrical phantoms (2 mL Eppendorf tubes) were prepared by suspending micron‐sized iron particles (MPIOs) (Compel, Bangs Laboratories; diameter [8.2 ± 0.6] μm, 10–90 percentile range = [7.8; 8.5] μm; 9.2 pg. iron per particle, 1000 particles/mL) in 1% agar gel. Because of their size and iron content, these particles produced signal voids similar to those created by iron‐labeled cells in vivo and in vitro.^18^

To study motion at very low velocities, an in‐house built rotating phantom system was used as described recently^20^ enabling rotation of phantoms inside the small animal scanner at constant angular velocities of as little as 1.46 × 10^−3^ rpm. Agar gel phantoms with embedded MPIOs were scanned static as a reference and then while rotating with maximal rotational speeds of 4.7 × 10^−2^ rpm translating to particle speeds of up to 1.5 mm/min. Individual particles were identified as signal voids and temporal blurring was assessed by quantifying signal loss (i.e., contrast) and void size of each particle as previously described.^20^ Briefly, signal loss was calculated as $\text{SL}=[\text{mean}({\text{signal intensity}}_{\text{enclosing area}})\text{–}\min(\text{signal}{\text{intensity}}_{\text{hypointense spot}})]/\text{mean}({\text{signal intensity}}_{\text{enclosing area}})$. To measure the void size, an image threshold was determined by averaging the minimum value and the background intensity. The void size was then quantified by counting the number of pixels below this threshold.

Subsequently, a velocity detection limit, defined as the velocity at which iron particles were not detectable anymore, was measured as previously described.^20^ Briefly, the change in signal loss ΔSL_i_ = SL_stat,i_ – SL_rot,i_ was calculated for all detected particles i, where SL_stat,i_ and SL_rot,i_ are the signal losses in the static and rotating phantom, respectively. Particles that were not observable anymore in the rotating phantom were referred to as “non‐visible” and their signal loss SL_rot,i_ was set to zero. For visible and non‐visible particles, respectively, linear regression of the velocity‐dependent change in signal loss was performed independently and the intersection of the two fits was declared as the velocity detection limit v_max_ ($\bar{\text{SLstat}}$). This calculation was repeated for varying initial signal losses SL_stat_ where only respective particles with an initial signal loss in the range of [SL_stat_ − 0.05; SL_stat_ + 0.05] were considered. The error of the maximum detectable velocity v_max_ was estimated through error propagation of the standard errors of the fit parameters from the respective fits. Following this procedure, the velocity detection limit was determined for the 2D Cartesian as well as the FS and CS 3D radial bSSFP, respectively. See Section 2.3 for details on the respective MRI sequence parameters.

Additionally, to assess differences in performance of FS and CS reconstructions in 3D radial bSSFP, only particles that were visible in both were analyzed and their velocity dependent relative change in signal loss was compared. This change was defined as the difference in signal loss between the two reconstruction modes divided by the average of the signal loss in the rotating case: relative change SL_i_ (FS, CS) = (SL_i_(CS) – SL_i_(FS))/((SL_i_(CS) + SL_i_(FS))/2) for all particles i.

### 2.2 In vivo experiments

Animal experiments were carried out according to local animal welfare guidelines and were approved by local authorities (ID: 81–02.04.2020.A194). Female BALB/c mice (*n* = 3 for 2D Cartesian bSSFP) or female C57/BL6 mice (*n* = 2 for 3D radial bSSFP) were obtained from Charles River Laboratories and housed under a 12 h light–dark cycle and provided with food and water ad libitum.

Cells were labeled in vivo by i.v. injection of 1.5 mmol Fe/kg body weight (bw) of Ferucarbotran (Resovist, Bayer AG; 0.5 mmol Fe/mL) (*n* = 2) or 1.5 mmol Fe/kg bw (*n* = 1) or 0.95 mmol Fe/kg bw (n = 1) of FeraSpin functionalized with Cy3 (Viscover, nanoPET Pharma; 0.18 mmol Fe/mL) via the tail vein. In vivo time‐lapse MRI of the brain was performed 24 h after injection to ensure clearing of free iron particles from the blood stream.^8^, ^18^

Mice received either an i.p. induction dose with ketamine (K) (80 mg/kg), xylazine (X) (8 mg/kg), and acepromazine (0.5 mg/kg) and KX (40/4 mg/kg/h) as a continuous i.p. infusion (3D radial bSSFP; *n* = 2) or were anesthetized with 1% to 1.5% isoflurane (2D Cartesian bSSFP; *n* = 2) in 1 L per minute of oxygen and compressed air (20:80) and positioned in a warmed animal bed. The head was fixed by ear plugs and bite bar. Respiration and temperature were continuously monitored and kept in the physiological range.

To minimize banding artifacts by improving shimming conditions, mice heads were shaved, and ultrasound‐gel and a ˜1 mm thick 1% agarose gel sheet was placed on top of the head for 2D Cartesian bSSFP imaging.

Individual cells were visually identified as hypointense spots and manually tagged using FIJI.^27^ Coordinates were linked to trajectories with an adapted cell tracking tool that uses the corresponding part of the MATLAB (The Mathworks) implementation by Blair and Dufresne^28^ of the interactive data language particle tracking code of Crocker et al.^29^, ^30^ and cell motion velocities were determined. To avoid mistaking small vessels for cells, stationary features visible in all timeframes were not counted.

### 2.3 MRI acquisition and image reconstruction

All MRI experiments were performed on a 9.4 T 94/20 Bruker Biospec equipped with cryogenic probe, and operated under Paravision 6.0.1 (Bruker Biospin).

#### 2.3.1 Two dimensional Cartesian bSSFP

The scan parameters for 2D Cartesian bSSFP imaging were: TE/TR: 6.0/12 ms, flip angle (FA): 30°, averages: 32, acquisition bandwidth: 50 000 Hz, acquisition/image matrix: 256 × 256, in‐plane resolution: 59 × 59 μm^2^, one slice of 300 μm, scan time per timeframe: 1:42.85 min.

To improve single‐cell detection in vivo 2D Cartesian bSSFP imaging, retrospective contrast enhancement was performed by calculating a four‐times finer imaging grid through zero‐filling and a minimum intensity projection of the 4 × 4 sample points onto the native resolution.^31^

For comparison in phantom measurements, the full‐brain Cartesian time‐lapse MRI protocol, a $T_{2}^{*}$‐weighted GRE sequence, was used as presented in previous studies.^18^, ^19^ The scan parameters were: TE/TR: 8.0/645 ms, FA: 60°, averages: 4, acquisition bandwidth: 50 000 Hz, acquisition matrix: 180 × 192, image matrix: 180 × 256, in‐plane resolution: 67 × 55 μm^2^, 38 contiguous slices covering the whole brain, slice thickness: 300 μm, scan time per timeframe: 8:15.36 min.

#### 2.3.2 Three dimensional radial imaging

For 3D radial bSSFP in time‐lapse MRI a dual half‐echo balanced ultra‐short echo time sequence^32^ with a center‐out—center‐in k‐space trajectory and an interleaved ordering scheme was used. Two half‐echoes are acquired, one at the beginning and one at the end of TR, that can be reconstructed separately and combined subsequently. The scan parameters were: TE_1_/TE_2_/TR: 0.65/3.33/4 ms, FA: 45°, averages: 1, acquisition bandwidth: 100 000 Hz, number of spokes: 120 188, interleaved factor *f*: 20, sampling points per spoke: 259, image matrix: 196 × 196 × 196, spatial resolution: 77 × 77 × 77 μm^3^, scan time per FS timeframe: 8:00.75 min, repetitions: 10, non‐selective 32 μs pulse, phase cycling between consecutive spokes (0, π).

Data was reconstructed using compute unified device architecture‐based graphics processing units non‐uniform fast Fourier transform.^33^ To compensate for variable density of sampling points, a ramp filter was applied to each spoke. In addition, during gradient ramping, sample density was estimated based on the derivative of the absolute distance of sampling points to the origin. The two separate echo images $I_{1}$ and $I_{2}$ were reconstructed independently and combined by a weighted combination, originally proposed for combining multiple bSSFP images with different RF phase increments,^34^ with a weighting factor of *p* = −0.5:

$$I_{\text{comb}}={\left|{\left|I_{1}\right|}^{p}\;I_{1}+{\left|I_{2}\right|}^{p}\;I_{2}\right|}^{\left(\frac{1}{1+p}\right)}$$

Furthermore, because of the interleaved ordering scheme, all acquired spokes can be divided into *f* US subframes defined by the interleaved factor *f*, for instance here, 20 subframes with 6009 spokes each equally distributed over 3D k‐space (360°). Grouping a larger number of spokes leads to a smaller US ratio. To ensure uniform sampling of k‐space, this cannot be chosen arbitrarily, but rather subframes have to be combined.

To enhance image quality, the US datasets were additionally reconstructed with CS using 3D golden‐angle‐radial sparse parallel reconstruction^35^, ^36^ implemented in MATLAB. A weighting parameter λ of 1% was applied for phantom and of 5% for in vivo experiments.

Three dimensional representation was performed using maximum intensity projection (MIP) on the inverted images with Horos (https://horosproject.org), a free and open source code software program that is distributed free of charge under the LGPL license and sponsored by Nimble d/b/a Purview in Annapolis, MD.

### 2.4 Image analysis

SNR was measured as mean signal intensity in regions of interest (ROIs) divided by the SD of noise. SNR per unit time was calculated as SNR per scan time. Representative slices were chosen and ROIs were placed in the mouse brain cortex or at different positions within the phantom and a mean SNR was calculated. CNR was defined as the difference between the minimum signal intensity of a signal void and the mean signal intensity in the enclosing area divided by the SD of the signal intensity in the enclosing area. To account for the Rayleigh distribution of the background noise in magnitude images, noise was multiplied by the correction factor √ 2/(4 − *π*).

Results shown are means ± standard error of the mean.

## 3 RESULTS

### 3.1 Two dimensional Cartesian bSSFP

**FIGURE 1:**
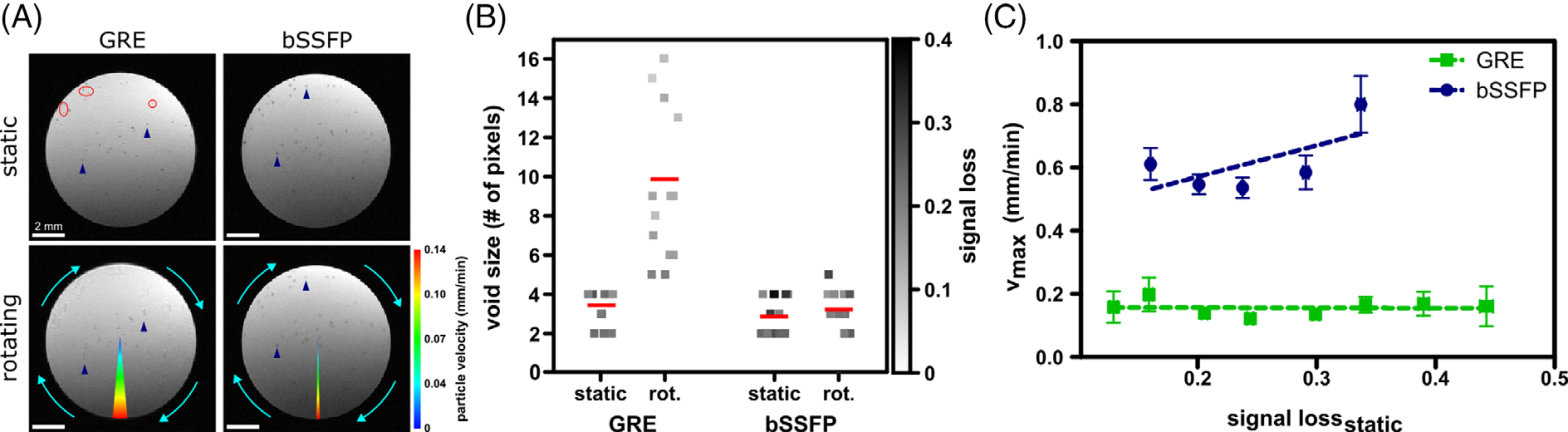
Rotating phantom measurements comparing 2D Cartesian balanced SSFP (bSSFP) and gradient echo (GRE) imaging in time‐lapse MRI. (A) An agarose phantom containing micron‐sized iron particles (MPIOs) was scanned in a static position as reference and then while rotating (here, at 4.4 × 10^−3^ rpm) using both sequences. Exemplary measurements are shown. The circle segments indicate how far the phantom rotated during the respective time frame. The colormap signifies the particle velocity dependent on the radial distance from the axis of rotation. Single iron particles were resolved as hypointense spots. In the rotating phantom, temporal blurring was observed as decreased signal loss and elongated shapes (blue arrowheads). This effect was stronger in GRE images compared to bSSFP acquisition. Additionally, in GRE images, some particles disappeared once the phantom is rotating (red circles), whereas in bSSFP images all iron particles remained visible at the same rotational speed. (B) Comparison of void size and signal loss of 14 particles detectable in the rotating phantom using both sequences respectively, confirmed stronger temporal blurring in GRE than bSSFP. Individual data points represent single particles with the intensity indicating signal loss. Red horizontal bars are group means. (C) Using the rotating phantom system, the maximum detectable velocities v_max_ of bSSFP (blue) and GRE (green) were derived for different given initial values of signal loss SL_stat_.

First, the capability of 2D Cartesian bSSFP imaging to detect single iron particles was assessed in phantoms. In the stationary case (Figure 1A, upper row), a mean SNR of 69.4 ± 1.1 (*n* = 3 ROIs) was achieved that was comparable to GRE images (mean SNR of 88.0 ± 4.8), whereas, considering the scan time, the SNR per unit time in bSSFP images of (40.8 ± 0.6) min^−1^ was notably higher than in GRE images ([10.7 ± 0.6] min^−1^). The detected hypointensities resembled those observed using GRE. Twenty‐three detected single MPIOs generated signal voids with a similar mean signal loss in bSSFP (0.24 ± 0.01) and GRE images (0.21 ± 0.01).

Under rotating conditions (Figure 1A, lower row; Video S1), single particles exhibited temporal blurring as decreased signal loss and elongated shapes. At the same rotational speed of 4.4 × 10^−3^ rpm, of 23 particles, detected in one imaging slice, 9 particles (39%) disappeared in GRE images, whereas all particles remained visible in bSSFP images. Blurring effects appeared stronger in GRE compared to bSSFP images. Particle sharpness quantified by signal loss and void size corroborated this perception (Figure 1B): 14 detected particles, that remained visible in the rotating phantom in GRE and bSSFP images, showed only a slight reduction of signal contrast (i.e., signal loss) from 0.26 ± 0.02 in the static to 0.23 ± 0.01 in the rotating phantom and remained relatively small with a mean void size of (3 ± 0) pixel under both conditions when bSSFP imaging was used. Contrarily, in GRE images, a strong decrease in signal loss from 0.23 ± 0.01 to 0.13 ± 0.01 was evident and hypointensities were substantially bigger in the rotating ([10 ± 1] pixel) compared to the static phantom ([3 ± 0] pixel).

Particle visibility was affected by velocity, but also by the contrast in the static case. Velocity detection limits of 2D Cartesian bSSFP in single‐cell tracking of up to (0.80 ± 0.09) mm/min were determined in vitro for the investigated signal losses (Figure 1C, Table S1). These were higher compared to GRE (0.1–0.2 mm/min).^20^ The fastest detectable particle in GRE images had a speed of 0.23 mm/min, whereas in bSSFP images a particle as fast as 1.0 mm/min was tracked.

**FIGURE 2:**
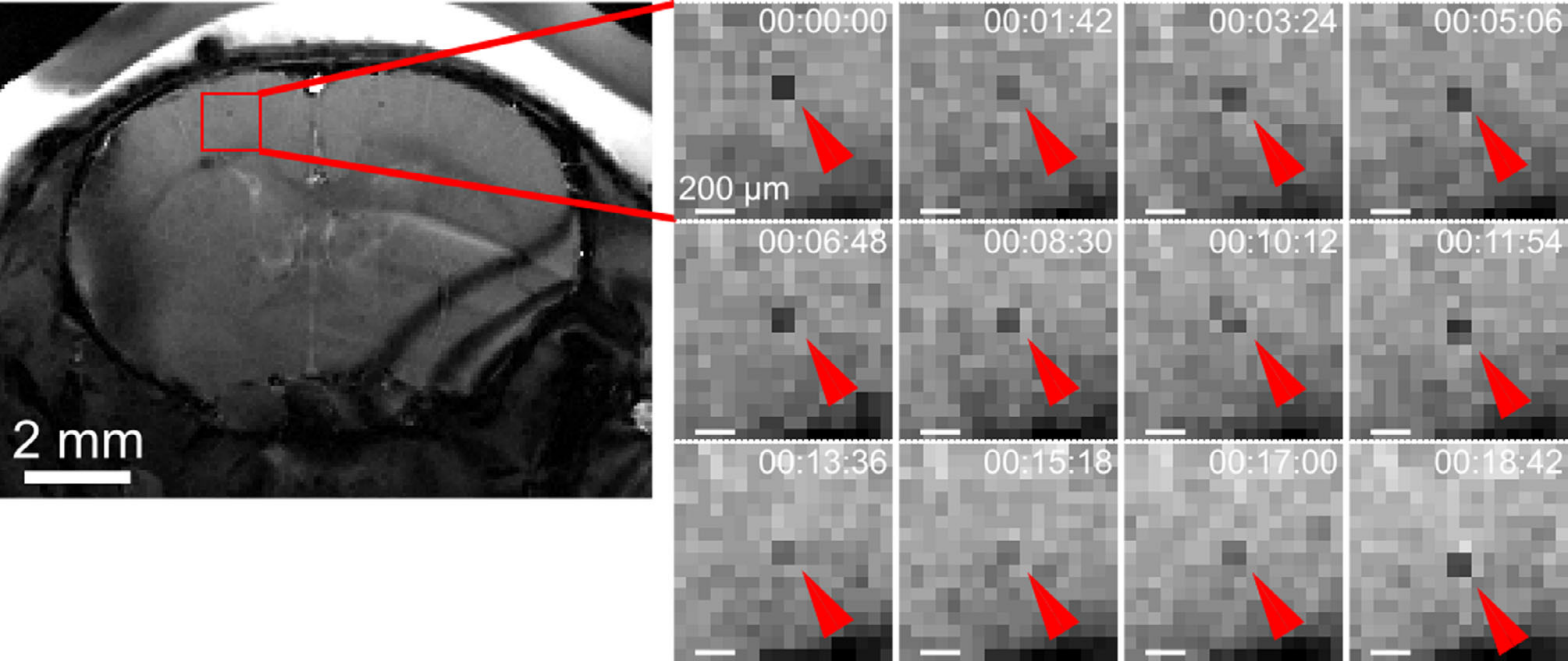
In vivo time‐lapse MRI using 2D Cartesian balanced SSFP acquisition. Image details (indicated by the red rectangle) show consecutive timeframes with an example of a single iron‐labeled cell (red arrowheads) moving across several voxels.

Because 2D Cartesian bSSFP imaging was successful in resolving single static and moving MPIOs in phantoms, the sequence was applied to in vivo time‐lapse MRI in the mouse brain. Again, single iron‐labeled immune cells produced similar hypointensities as with the established 2D Cartesian GRE time‐lapse MRI sequence (Figure 2, Video S2). Additional contrast enhancement through zero‐filling improved image quality and facilitated single‐cell detection in 2D Cartesian bSSFP imaging (Figure S1). While some cells only appeared in a single time‐frame, others showed distinct movement. In *n* = 2 mice, a total of 38 cells moving across several voxels in at least two consecutive timeframes were detected with a mean velocity of (19.2 ± 1.6) μm/min with the fastest detectable cell moving with a speed of 50 μm/min. Off‐resonance banding artifacts occurred yet did not hinder single‐cell detection substantially because they were locally constrained. A reduction of TR was limited by gradient performance and required larger pixel size, which decreased single‐cell visibility, as assessed in phantom measurements (Figure S2A,B), and did not remove banding artifacts in in vivo images (Figure S2C).

### 3.2 3D radial bSSFP

Although 2D Cartesian bSSFP imaging allows for single‐cell detection at high temporal resolution, it is limited to a single imaging slice. To increase the imaging volume, a 3D radial sampling scheme was implemented, allowing total brain coverage at isotropic resolution.

**FIGURE 3:**
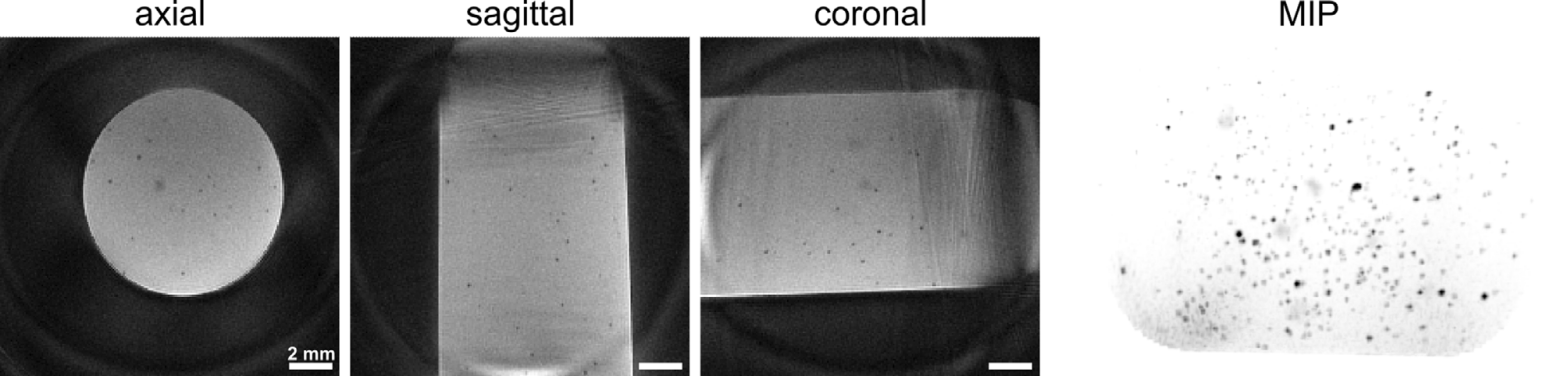
Phantom measurements using fully sampled (FS) 3D radial sampling in balanced SSFP (bSSFP) imaging. Exemplary images of an agarose phantom containing micron‐sized iron particles (MPIOs) are shown. Single iron particles were identified as signal voids in all three dimensions (axial, sagittal, and coronal) and a 3D representation was achieved through maximal intensity projection (MIP).

As a first step, to test its performance on single‐cell imaging, the method was applied in vitro to agar phantoms with iron particles. In all three dimensions, axial, sagittal, and coronal, hypointense spots originating from single MPIOs with comparable appearance as in GRE and 2D Cartesian bSSFP images were identified (Figure 3). Owing to the isotropic resolution, a 3D representation of the phantoms was generated using MIP visualizing the 3D distribution of iron particles within the agar phantom (Figure 3). The implemented weighted combination of both echoes performed best in terms of SNR, signal loss, CNR, and overall image quality compared to the individual echo images and a sum‐of‐squares combination (Figure S3).

**FIGURE 4:**
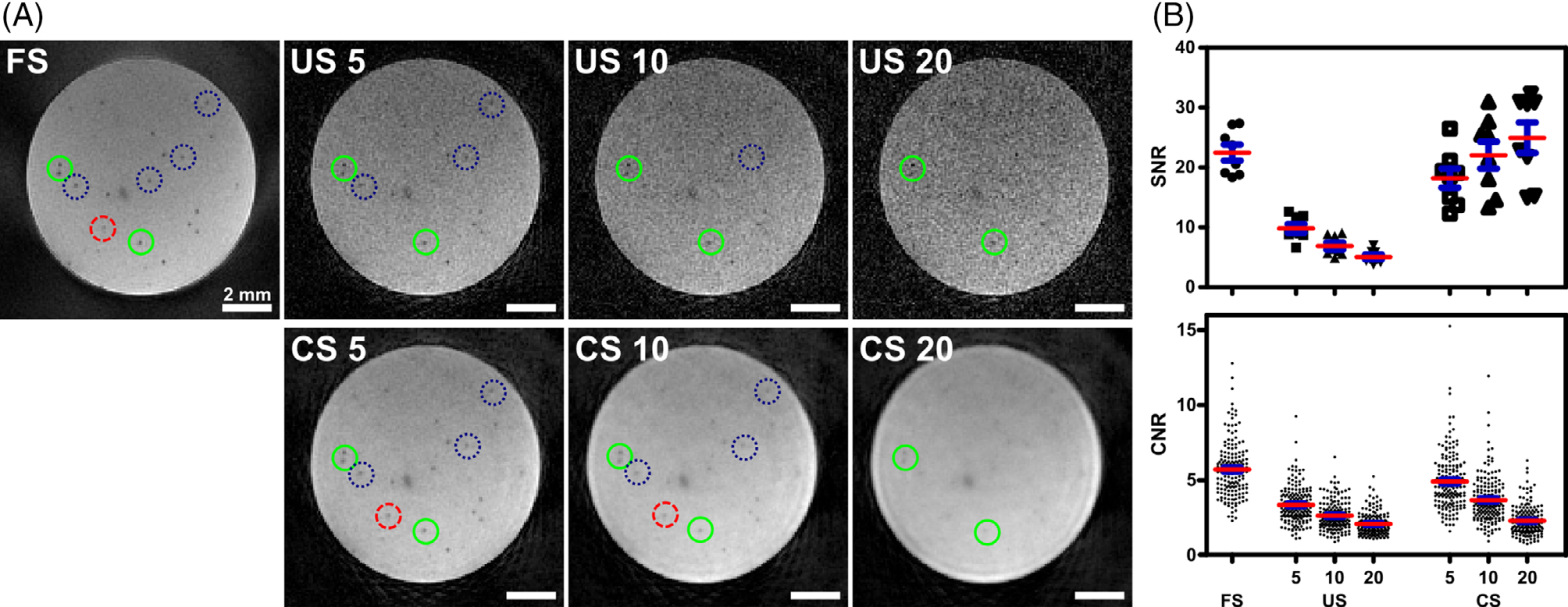
Phantom measurements using 3D radial sampling with interleaved ordering scheme and compressed sensing reconstruction in balanced SSFP (bSSFP) time‐lapse MRI at different acceleration factors. (A) Exemplary images of an agarose phantom containing micron‐sized iron particles (MPIOs) are shown for the fully sampled (FS) reconstruction as well as for undersampled (US) reconstruction at different acceleration factors defined by the number of subsets (5, 10, and 20, respectively). The lower row shows the corresponding frames with additional compressed sensing (CS) reconstruction. Single iron particles were identified as signal voids in all images. High contrast particles remained visible regardless of the reconstruction mode and acceleration factor (green circles). However, low contrast features were not detected in accelerated images, especially at high acceleration factors (blue dotted circles). CS improved image quality and facilitated particle detection. Here, particles were recovered that were not discernible in US images (red dashed circle). (B) These observations were corroborated by measuring SNR of the images and contrast‐to‐noise ratios (CNR) of hypointense spots as indicators for image quality for the different reconstruction modes. Data points represent individual region‐of‐interests (ROI) and single particles, respectively, red horizontal bars group means with standard error of the mean (SEM) in blue.

With the aim of increasing the temporal resolution, an interleaved ordering scheme was implemented, allowing the retrospective reconstruction of images at variable temporal resolution. Compared to conventional sequential sampling, minor imaging artifacts because of eddy currents arose (Figure S4). However, they were restricted to the edge of the volume and, therefore, did not disturb the overall image as opposed to alternative sampling schemes such as (tiny) golden angle sampling^37^, ^38^ (Figure S4). Phantom images were reconstructed using all acquired spokes (FS) or using US subsets creating 5, 10, and 20 subframes with corresponding temporal resolutions per timeframe of 01:36.15 min, 00:48.075 min, and 00:24.036 min, respectively. Additionally, CS reconstruction was applied to the US subsets. In all three reconstructions modes, FS as well as US and CS at different acceleration factors, single MPIOs were detected as hypointensities a few pixels in size (Figure 4A). However, image quality visibly decreased with increasing acceleration hampering particle detection in US and CS images. Although high contrast particles were discernible in all reconstruction modes, those with lower contrast were not detected in accelerated images, especially in US images because of the augmented noise. CS improved image quality compared to US, resulting in smooth images and allowed for the recovery of some particles hidden in US images. However, for high acceleration factors, again most iron particles could not be detected (Figure 4A). Quantification of SNR and CNR confirmed these observations (Figure 4B). In FS images of the static phantom, a mean SNR of 23 ± 1 (*n* = 8 ROIs) and a mean CNR of 5.7 ± 0.1 (*n* = 175 hypointensities) was achieved. In US images, the SNR decreased with 1USfactor to 10 ± 1, 7 ± 1, and 5 ± 0. CNR decreased to 3.4 ± 0.1, 2.6 ± 0.1, and 2.1 ± 0.1 for the acceleration factors of 5, 10, and 20, respectively. CS was able to push the SNR to 18 ± 2, 22 ± 2, and 25 ± 3 for the three different acceleration factors, respectively. CNR also decreased with increasing temporal resolution (4.9 ± 0.1, 3.7 ± 0.1, and 2.3 ± 0.1). Nevertheless, for all three acceleration factors the CNR in CS was lower than in FS images, but higher compared to the corresponding US images. Overall, an acceleration factor of 5 with CS reconstruction demonstrated the best results in terms of image quality, particle detectability, and temporal resolution and was, therefore, chosen for any further reconstruction and analysis.

**FIGURE 5:**
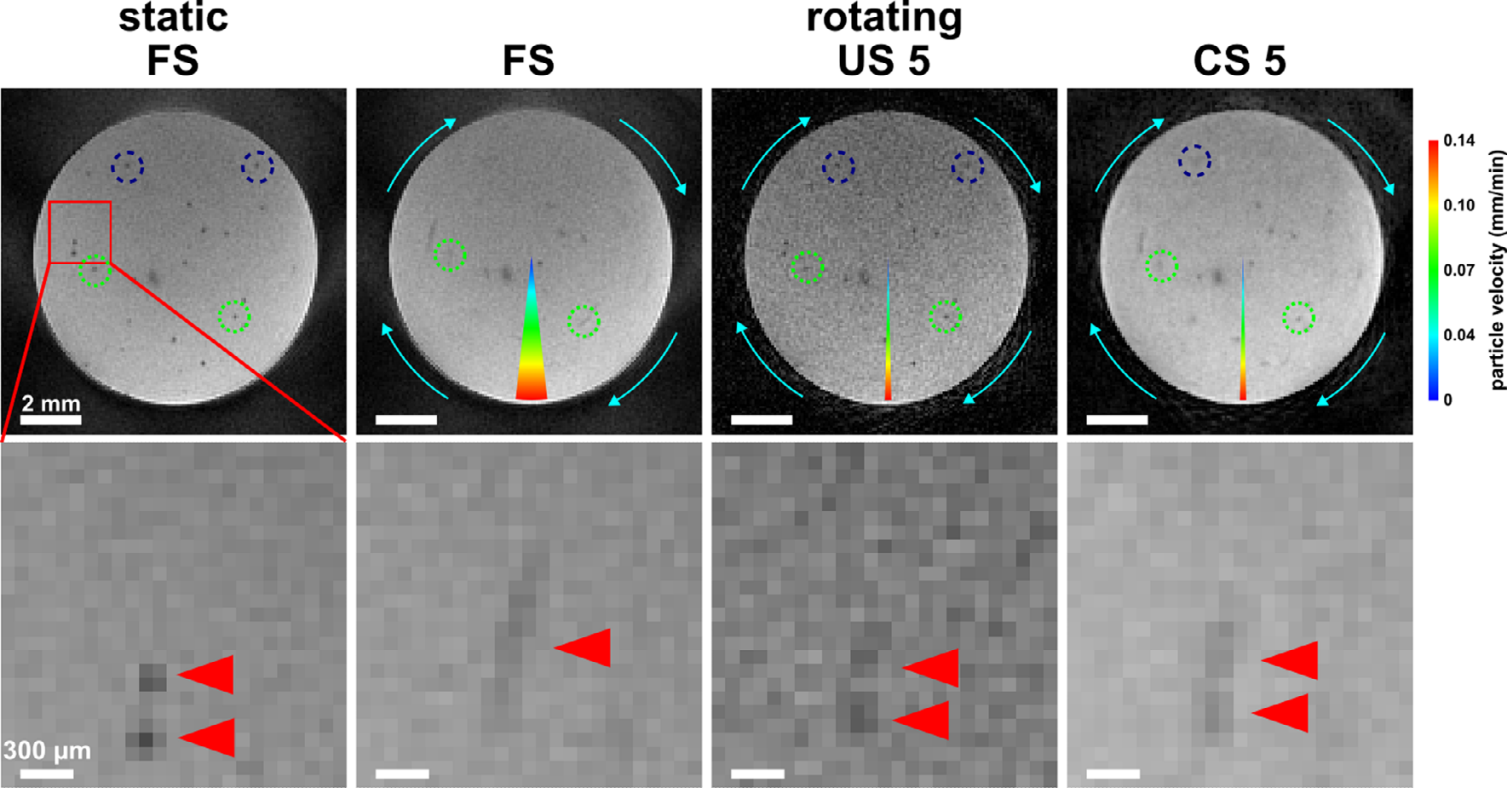
Rotating phantom measurements using 3D radial sampling with interleaved ordering scheme and compressed sensing reconstruction in balanced SSFP (bSSFP) time‐lapse MRI. Exemplary measurements of an agarose phantom containing micron‐sized iron particles (MPIOs) scanned in a static position as reference (first column) and then while rotating (here, at 4.4 × 10^−3^ rpm; second to fourth column) are shown. Images were reconstructed with all acquired spokes (fully sampled [FS]), and using only a subset with one fifth of the acquired spokes without (undersampled [US 5]) and with additional compressed sensing (CS 5). In the upper row, circle segments indicate how far the phantom rotated during the respective time frame. The colormap signifies the particle velocity dependent on the radial distance from the axis of rotation. Single iron particles were resolved as small hypointensities. Once the phantom rotated during the acquisition, particles became blurred (green dotted circles) or faded (blue dashed circles) in FS images. Because of the higher temporal resolution, temporal blurring was weaker in US and CS images (green dotted circles) and some particles were recovered (blue dashed circles). In the lower row, image details (indicated by the red square) show an example of two particles (red arrowheads) that could not be resolved separately in FS images of the rotating phantom because of severe blurring effects. However, using US and CS reconstruction two particles were clearly distinguished from another.

Next, to assess whether 3D radial bSSFP is able to image moving iron particles, rotating phantom measurements were performed (Figure 5, Video S3). Similar to 2D Cartesian GRE and bSSFP, temporal blurring was evident as reduced signal loss (i.e., contrast), stretched shapes, and the disappearance of particles at high velocities. In US and CS images, particles exhibited less blurring because of the higher temporal resolution, and some particles were even recovered. In fact, of 829 representatively detected particles in the 3D imaging volume in the static case, 361 (43.5%) were not visible in the rotating phantom regardless of the reconstruction mode, 257 (31.0%) were detectable in the FS reconstruction, whereas 459 (55.4%) were discernible in the accelerated CS images. Furthermore, in some cases temporal blurring in FS images under rotating conditions impeded differentiation of neighboring particles, which were clearly distinguishable in the static phantom. Contrarily, in US and CS images of the rotating phantom, distinct hypointense spots were visible enabling the separation of the two particles (Figure 5, lower row).

**FIGURE 6:**
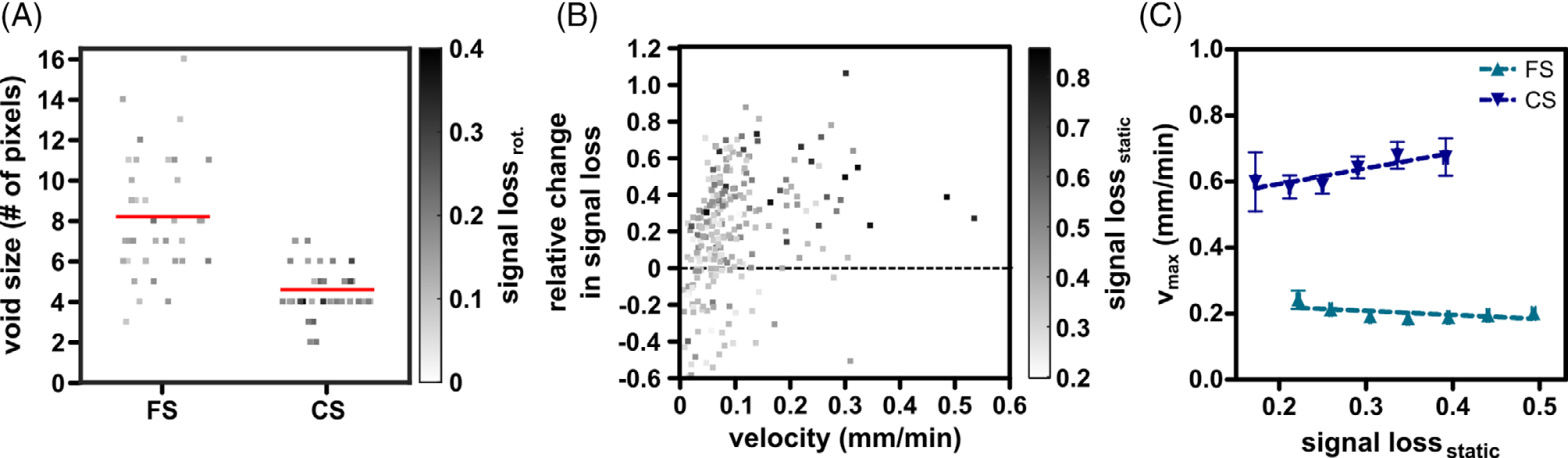
Improvement of particle visibility of compressed sensing (CS) compared to fully sampled (FS) reconstruction in 3D radial balanced SSFP (bSSFP) imaging. (A) For both image reconstruction methods, FS, and CS, temporal blurring of 38 particles with similar velocity ([68 ± 4] μm/min) was quantified by calculating void size and signal loss in the rotating phantom. In each group, individual data points represent single particles with the dot intensity indicating the signal loss. Red horizontal bars are group means. (B) The velocity‐dependent relative change in signal loss was calculated as the difference in signal loss in the rotating phantom between the two images (CS–FS), divided by the average signal loss of the two methods. Individual data points represent single particles, and their intensity is given by the signal loss in the reference static phantom. A small fraction of particles showed a negative relative change, which indicated that CS performed worse than FS in terms of contrast (i.e., signal loss), most particles showed a positive change indicating that CS reconstruction improves the particle visibility through increased signal loss compared to FS reconstruction. (C) The maximum detectable velocity v_max_ was derived for FS and CS reconstruction at given initial values of signal loss SL_stat_.

Quantification of particle sharpness further corroborated the observed improvement of particle visibility of CS compared to FS images (Figure 6A). To reduce effects of variable velocities, 38 particles with similar speed ([68 ± 4] μm/min) visible in both reconstruction modes were assessed. While in the FS images, hypointensities were relatively big ([8 ± 0] pixel) with a low signal loss (0.13 ± 0.01), in CS their size decreased ([5 ± 0] pixel) and signal loss increased (0.19 ± 0.01) following conservation of signal. Moreover, fast‐moving particles (v >0.1 mm/min) particularly benefitted from the improved temporal resolution provided by CS as evidenced by the positive relative change in contrast (i.e., signal loss) between FS and CS (Figure 6B). Irrespective of the initial contrast, the signal loss was higher in CS than in FS images. Moreover, some slow‐moving cells (v < 0.1 mm/min) with low initial contrast showed reduced signal loss in CS images. Additionally, a velocity detection limit was measured based on the rotating phantom images (Figure 6C, Table S1). FS reconstruction achieved maximum detectable velocities of up to (0.20 ± 0.01) mm/min, whereas CS with an acceleration factor of 5 could increase it to (0.68 ± 0.04) mm/min. The fastest particles detectable had a speed of 0.54 mm/min in FS images and of 1.3 mm/min in CS images.

**FIGURE 7:**
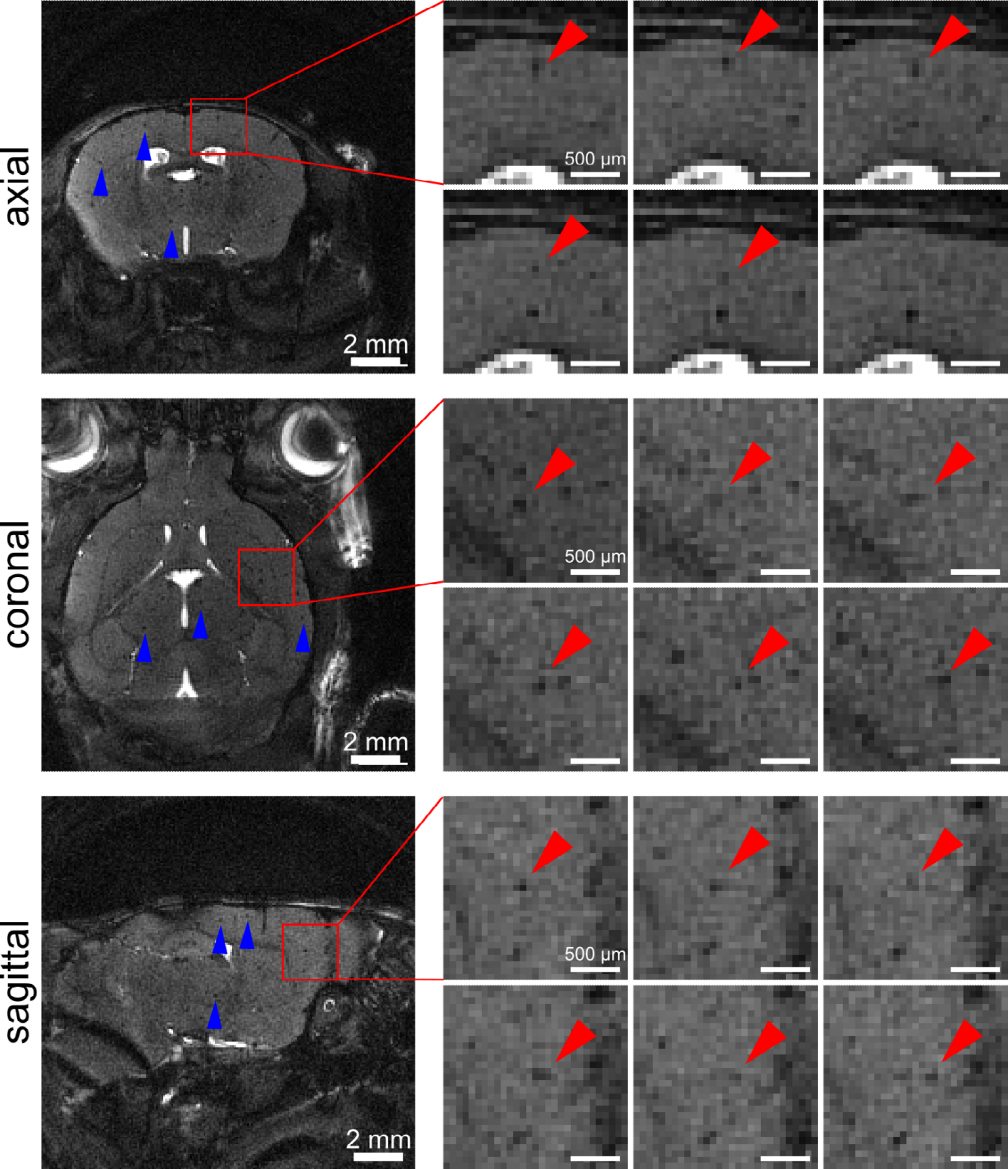
In vivo time‐lapse MRI using fully sampled (FS) 3D radial balanced SSFP (bSSFP) acquisition. In all three dimensions, single iron‐labeled cells were detected as hypointense spots (examples marked with arrowheads) in exemplary slices. Image details (indicated by the red rectangle) show consecutive timeframes with examples of singles cell (red arrowheads) moving across several voxels.

**FIGURE 8:**
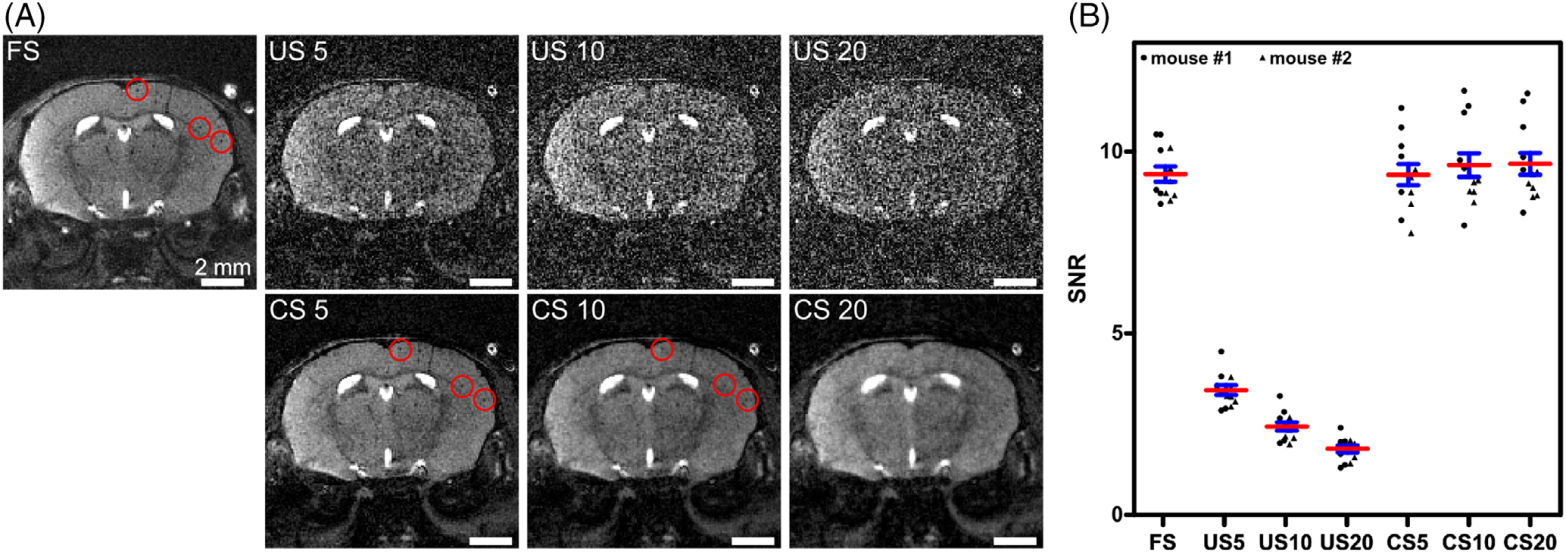
In vivo time‐lapse MRI using 3D radial sampling with interleaved ordering scheme and compressed sensing reconstruction in balanced SSFP (bSSFP) imaging. (A) Exemplary images of a mouse brain are shown for the fully sampled (FS) reconstruction as well as for undersampled (US) reconstruction at different acceleration factors defined by the number of subsets (5, 10, and 20, respectively). The lower row shows the corresponding frames with additional compressed sensing (CS) reconstruction. Single iron‐labeled cells were identified as signal voids. Red circles indicate examples of single cells visible in FS and CS images with fivefold or 10‐fold acceleration, but not discernible in any US images because of increased noise, nor in highly accelerated CS images. (B) SNR in FS, US, and CS images was calculated at the different acceleration factors for two mice in six regions‐of‐interest (ROIs) each. Data points represent individual ROI and red horizontal bars group means with standard error of the mean in blue.

**FIGURE 9:**
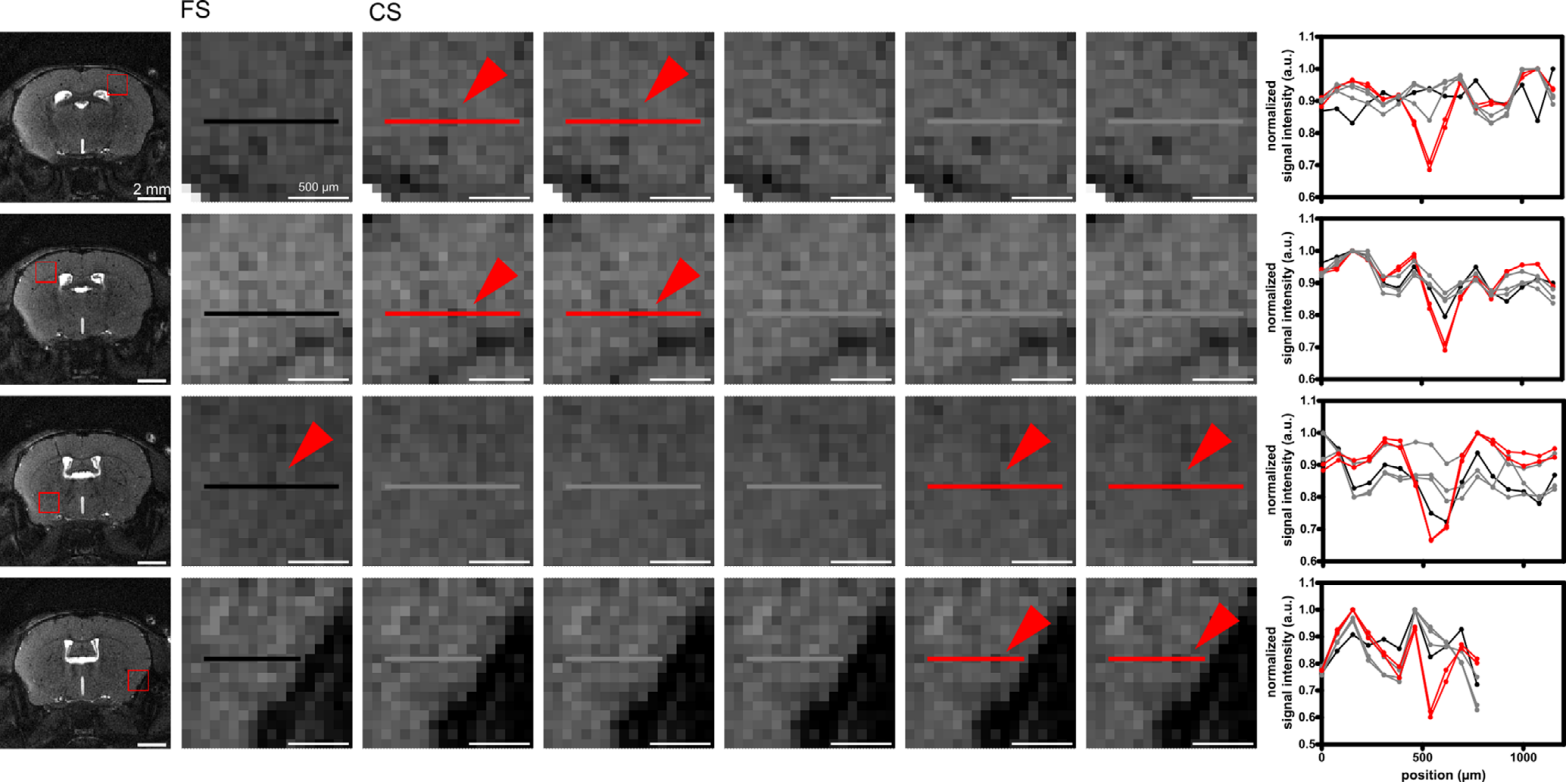
Additionally detected cells in in vivo single cell tracking using 3D radial balanced SSFP (bSSFP) imaging with compressed sensing (CS) reconstruction. Indicated by the red rectangle in representative slices at various positions in the mouse brain (first column), the image details of fully sampled (FS) images (second column) are compared with the corresponding accelerated CS subframes (third to seventh columns). These comparisons revealed examples of cells that were clearly identifiable in at least two subframes of the accelerated CS reconstruction but were either not visible (lines 1, 2, and 4) or barely visible (lines 3) in the FS images because of significant temporal blurring. Line plots, indicated by lines, illustrate the signal loss at the corresponding hypointense spots (eighth column).

Subsequently, in vivo time‐lapse imaging of mouse brains was performed using the 3D radial bSSFP sequence with interleaved sampling. Slight banding artifacts occurred, especially in the last timeframes. Yet, they were tolerable and single iron‐labeled cells were resolved in all three dimensions, axial, sagittal, and coronal, using all the acquired spokes (FS) (Figures 7 and 8A, Video S4). A mean SNR of 9.4 ± 0.2 was achieved (*n* = 2 mouse brains with *n* = 6 ROI each) (Figure 8B). In US images, noise increased drastically making single‐cell detection impossible (Figure 8A). SNR decreased with 1USfactor to 3.4 ± 0.1, 2.4 ± 0.1, and 1.8 ± 0.1 for acceleration factors of 5, 10, and 20, respectively (Figure 8B). CS yielded a mean SNR of 9.4 ± 0.3, 9.6 ± 0.3, and 9.7 ± 0.3, with increasing acceleration factor. Image quality improved enabling recovery of most cells hidden in US images (Figure 8A). However, for high acceleration factors, although images were of good quality, no cells could be detected (Figure 8A). As observed in phantoms, an acceleration factor of 5 yielded best results. Here, even additional cells were detected that were clearly visible in at least one of the five subframes following accelerated CS reconstruction, but they were not observed in the FS images (Figure 9).

## 4 DISCUSSION

In this work, bSSFP imaging was successfully applied to dynamic single‐cell tracking by time‐lapse MRI using 2D Cartesian and 3D radial sampling. Although 2D Cartesian bSSFP achieved imaging at high temporal resolution, but was limited to a single imaging slice, interleaved 3D radial sampling enabled whole‐brain coverage at isotropic resolution and allowed the retrospective reconstruction of FS, US, and CS images.

Both, in phantoms and in vivo, small signal voids a few pixels in size originating from single iron particles or single iron‐labeled intravascular cells with patrolling‐like velocity, respectively, were detected. Using both sequences, their appearance had high similarity to iron‐labeled monocytes assessed with $T_{2}^{*}$‐weighted GRE imaging.^11^, ^15^, ^18^, ^19^, ^20^ Even though for radial sampling, the periphery of k‐space is sampled with low density, single particle/cell detection was possible in all three dimensions and in the 3D representation using 3D radial bSSFP.

In rotating phantom images, motion‐dependent temporal blurring was mimicked and became visible as reduced contrast and elongated shapes. Although deformations do not change the particles' detectability in phantoms, in the mouse brain, they may be problematic, because cells can be easily mistaken for other brain structures such as vessels. Here, the advantage of bSSFP became evident. For 2D Cartesian, bSSFP temporal blurring was less pronounced than for GRE at the same rotational speed. For 3D radial sampling, US in combination with CS reconstruction was able to reduce blurring effects and even to recover particles hidden in FS images. Velocity detection limits of up to 0.8 mm/min of bSSFP were measured in vitro, fourfold higher than in GRE images. This limit was influenced by the static contrast and, therefore, the iron load of an individual cell, indicating the need for highly efficient cell labeling for single‐cell tracking by MRI.

In vivo, cells appeared for various time and length scales and the average velocity of moving cells aligned with the speed of patrolling monocytes reported in earlier time‐lapse MRI studies and intravital microscopy observations.^4^, ^18^

Two dimensional Cartesian bSSFP imaging was successful in resolving single iron‐labeled cells at high temporal resolution with high image quality, however, it was limited to a single slice hampering full organ coverage. In contrast to spoiled GRE imaging, where k‐space lines of multiple slices can be acquired within a given long TR, for bSSFP several slices need to be imaged sequentially resulting in extended scan times. Moreover, off‐resonance banding artifacts, common in bSSFP acquisition,^24^, ^34^, ^39^, ^40^ appeared in vivo, but were tolerable as they were localized and did not interfere with single‐cell detection. Attempts to reduce banding by shortening the repetition time were limited by gradient performance at the high spatial resolutions required for time‐lapse MRI. Longer TE also improved single‐cell detection by enhancing the contrast‐generating effect of iron.

To enhance the temporal resolution of 3D radial bSSFP in time‐lapse MRI, US schemes were evaluated against FS sequential sampling. Because of even k‐space coverage for an arbitrary number of spokes, golden‐angle sampling offers flexible data sorting and US, but introduces strong eddy current artifacts in bSSFP imaging because of large k‐space jumps.^37^, ^41^, ^42^ Although tiny‐golden angle patterns have shown potential to reduce these by minimizing angle increments,^37^ our implementation still experienced significant artifacts likely because of the need to sample a full sphere with center‐out acquisition. Interleaved sampling, although requiring a predetermined number of interleaves, proved superior by confining artifacts to the imaging edges, allowing effective particle and cell detection. A high interleaved factor in the proposed scheme enabled sufficient flexibility. Similar to 2D Cartesian^22^ and 2D radial^20^ sampling, the interleaved acquisition scheme allowed for retrospective reconstruction of FS and US images with and without CS. Although higher US ratios were possible, for single‐cell tracking, reduction of data for reconstruction to one‐fifth of all acquired spokes yielded best results in terms of image quality, temporal resolution, and single‐cell visibility. Within a scan time of <2 min per timeframe the whole mouse brain was imaged at high isotropic resolution. The accelerated image acquisition improved the detection of moving particles and cells notably compared to FS images owing to reduced temporal blurring and increased contrast. Distinct hypointense spots in US and CS allowed separation of particles that were indistinguishable in FS images and detection of additional particles and cells, which were previously hidden in FS images. In vivo, these are most likely cells that altered their velocity during their in‐plane movement, either by beginning to roll or by being carried away by the blood stream. Therefore, over the entire time frame, their average speed was not sufficiently low, but rather only during few subframes.

Albeit image quality of US reconstruction was sufficient to image single iron particles in phantoms, in vivo time‐lapse MRI suffered from increased noise preventing detection of cells in US images, indicating the need for advanced reconstruction methods such as CS. Further, phantom measurements showed that accelerated reconstructions were affected by the particles' motion and contrast characteristics. Slow‐moving particles, already detectable in FS images, did not benefit from acceleration. In fact, especially for those with low initial contrast, the signal loss even decreased in CS images compared to FS because of increased noise and the potential removal of low‐contrast features by CS.^43^ Moreover, high‐velocity particles showed improved visibility in accelerated CS images. Therefore, a combined analysis of FS and CS reconstructions is required for capturing both low‐contrast slow‐moving and fast‐moving cells.

Comparing phantom and in vivo measurements, single iron particles and iron‐labeled cells appeared similar with contrast depending on slice position and iron load. Expectedly, noise levels were lower in vitro and elongated shapes from temporal blurring were evident in phantoms, but not in vivo. Therefore, velocity detection limits derived in vitro are likely higher than in vivo. Moreover, phantom measurements were primarily restricted to in‐plane motion, whereas in vivo, cells exhibit movement across all three dimensions.

Overall, bSSFP imaging improved the temporal window of time‐lapse MRI. Yet, rolling monocytes, involved in immune responses and linked to velocities ˜2.4 mm/min, remain invisible for time‐lapse MRI. To potentially capture those as well, even shorter scan times and a higher contrast of single cells, which may be achieved by more efficient cell labeling, are required. Therefore, optimal k‐space sampling and image reconstruction will be explored in the future, for example, in combination with deep learning and compressed sensing in 2D Cartesian bSSFP. Additionally, cell labeling efficiency will be improved to more effectively capture single cells and extended to non‐phagocytic cell types, such as T‐cells, potentially providing detailed insights into the adaptive immune system.

## 5 CONCLUSIONS

bSSFP acquisition was successfully implemented for time‐lapse MRI, enabling improved single‐cell tracking. Both single iron particles in rotating phantoms and iron‐labeled cells in vivo can be followed dynamically. The 2D Cartesian bSSFP approach enhanced temporal resolution and reduced blurring effects, yet it was restricted to single‐slice imaging. In contrast, 3D radial sampling provided full organ coverage with high isotropic spatial resolution within feasible scan times. Leveraging accelerated acquisition techniques, such as radial US and CS reconstruction, effectively improved temporal fidelity and emphasized cellular detail. The achieved velocity detection limit of time‐lapse MRI was advanced to 0.8 mm/min, fourfold higher compared to GRE imaging.

## Supporting information


**Table S1.** Velocity detection limits of balanced steady‐state free precession (bSSFP) imaging in single‐cell tracking by time‐lapse MRI. The maximum detectable speeds v_max_ were measured for varying initial signal losses in the static reference image for 2D Cartesian sampling as well as for fully sampled (FS) and undersampled compressed sensing (CS) reconstruction in 3D radial sampling.
**Figure S1.** Retrospective contrast enhancement by zero‐filling (CE ZF).
**Figure S2.** Effect of the echo time (TE) and repetition time (TR) on 2D Cartesian balanced steady‐state free precession (bSSFP) imaging in time‐lapse MRI.
**Figure S3.** Comparison of first $I_{1}$ and second echo $I_{2}$, weighted (WC) ($I_{\mathrm{WC}}={\left|{\left|I_{1}\right|}^{p}\;I_{1}+{\left|I_{2}\right|}^{p}\;I_{2}\right|}^{\left(\frac{1}{1+p}\right)};p=-0.5$) and sum‐of‐squares (SOS) combination $\left(I_{\mathrm{sos}}=\left|\sqrt{{I_{1}}^{2}+{I_{2}}^{2}}\right|\right)$.
**Figure S4.** Eddy current artifacts in 3D radial balanced steady‐state free precession (bSSFP) for different sampling schemes.

**Video S1.** Rotating phantom measurements using 2D Cartesian balanced steady‐state free precession (bSSFP) acquisition in time‐lapse MRI. Single iron particles were identified as hypointense spots in the static (left) and rotating phantom (right). In the rotating case, 13 consecutive timeframes are shown. The rotational speed was 4.4 × 10^−3^ rpm with resulting particle speeds of up to 0.14 mm/min at the edge of the phantom. Slight temporal blurring of moving particles was observed as decreased signal loss and elongated shapes.

**Video S2.** Example of in vivo time‐lapse MRI using 2D Cartesian balanced steady‐state free precession (bSSFP) acquisition. Time‐lapse MRI video of one slice with 20 timeframes is shown. An acquisition time of 1 min 42 s per timeframe resulted in a total scan time of 34 min. Single cells were resolved as hypointense spots and were followed dynamically (red arrowheads).

**Video S3.** Improvement of the temporal resolution in in vitro time‐lapse MRI using an interleaved 3D radial acquisition scheme and CS reconstruction in balanced steady‐state free precession (bSSFP) imaging. Ten consecutive full timeframes and the corresponding 50 subframes of the accelerated reconstructions are shown. The rotational speed was 7.32 × 10^−3^ rpm. In the FS images (left), temporal blurring of moving particles was observed. In the US reconstruction (middle), motion distortion decreased, however noise increased. CS (right) improved image quality while keeping the high temporal resolution resulting in reduced temporal blurring compared to FS images.

**Video S4.** Example of in vivo time‐lapse MRI using 3D radial balanced steady‐state free precession (bSSFP) acquisition with interleaved ordering scheme. Time‐lapse MRI video of an exemplary axial slice with 10 fully sampled (FS) timeframes and the corresponding 50 subframes of the accelerated reconstructions without (US) and with compressed sensing (CS) is shown. An acquisition time of 8 min per FS timeframe resulted in a total scan time of 80 min. Single cells were resolved as hypointense spots and were followed dynamically in FS and CS images (red arrowheads).
