# Supplementary material for: Boosting the velocity detection limit of 3D single‐cell tracking time‐lapse MRI by balanced SSFP imaging
Source: Magn Reson Med. 2025 May 23;94(3):1152–65. doi: 10.1002/mrm.30553 (PMC12202727; doi:10.1002/mrm.30553)
Supplement: Supplementary file 1 — Table S1. Velocity detection limits of balanced steady‐state free precession (bSSFP) imaging in single‐cell tracking by time‐lapse MRI. The maximum detectable speeds vmax were measured for varying initial signal losses in the static reference image for 2D Cartesian sampling as well as for fully sampled (FS) and undersampled compressed sensing (CS) reconstruction in 3D radial sampling. Figure S1. Retrospective contrast enhancement by zero‐filling (CE ZF). Figure S2. Effect of the echo time (TE) and repetition time (TR) on 2D Cartesian balanced steady‐state free precession (bSSFP) imaging in time‐lapse MRI. Figure S3. Comparison of first I1 and second echo I2, weighted (WC) (IWC=I1pI1+I2pI211+p;p=−0.5) and sum‐of‐squares (SOS) combination (Isos=I12+I22). Figure S4. Eddy current artifacts in 3D radial balanced steady‐state free precession (bSSFP) for different sampling schemes. [file MRM-94-1152-s001.docx]

# Supporting Information

| **2D Cartesian bSSFP** | | **3D radial bSSFP** | | | |  |
| --- | --- | --- | --- | --- | --- | --- |
|  |  | **FS** | | **CS** | |  |
| **initial signal loss** | **v_max_ (mm/min)** | **initial signal loss** | **v_max_  (mm/min)** | **initial signal loss** | **v_max_ (mm/min)** | |
| 0.160 ± 0.002 | 0.61 ± 0.05 | 0.222 ± 0.002 | 0.24 ± 0.03 | 0.172 ± 0.002 | 0.60 ± 0.09 | |
| 0.201 ± 0.002 | 0.55 ± 0.03 | 0.259 ± 0.002 | 0.21 ± 0.01 | 0.212 ± 0.001 | 0.58 ± 0.04 | |
| 0.238 ± 0.002 | 0.54 ± 0.03 | 0.305 ± 0.001 | 0.19 ± 0.01 | 0.250 ± 0.001 | 0.59 ± 0.03 | |
| 0.291 ± 0.003 | 0.59 ± 0.05 | 0.348 ± 0.001 | 0.19 ± 0.01 | 0.291 ± 0.001 | 0.64 ± 0.03 | |
| 0.337 ± 0.003 | 0.80 ± 0.09 | 0.395 ± 0.001 | 0.19 ± 0.01 | 0.336 ± 0.002 | 0.68 ± 0.04 | |
|  |  | 0.441 ± 0.002 | 0.19 ± 0.01 | 0.392 ± 0.003 | 0.67 ± 0.06 | |
|  |  | 0.493 ± 0.002 | 0.20 ± 0.01 |  |  | |

**Table S1.** Velocity detection limits of balanced steady-state free precession (bSSFP) imaging in single-cell tracking by time-lapse MRI. The maximum detectable speeds v_max_ were measured for varying initial signal losses in the static reference image for 2D Cartesian sampling as well as for fully sampled (FS) and undersampled compressed sensing (CS) reconstruction in 3D radial sampling.


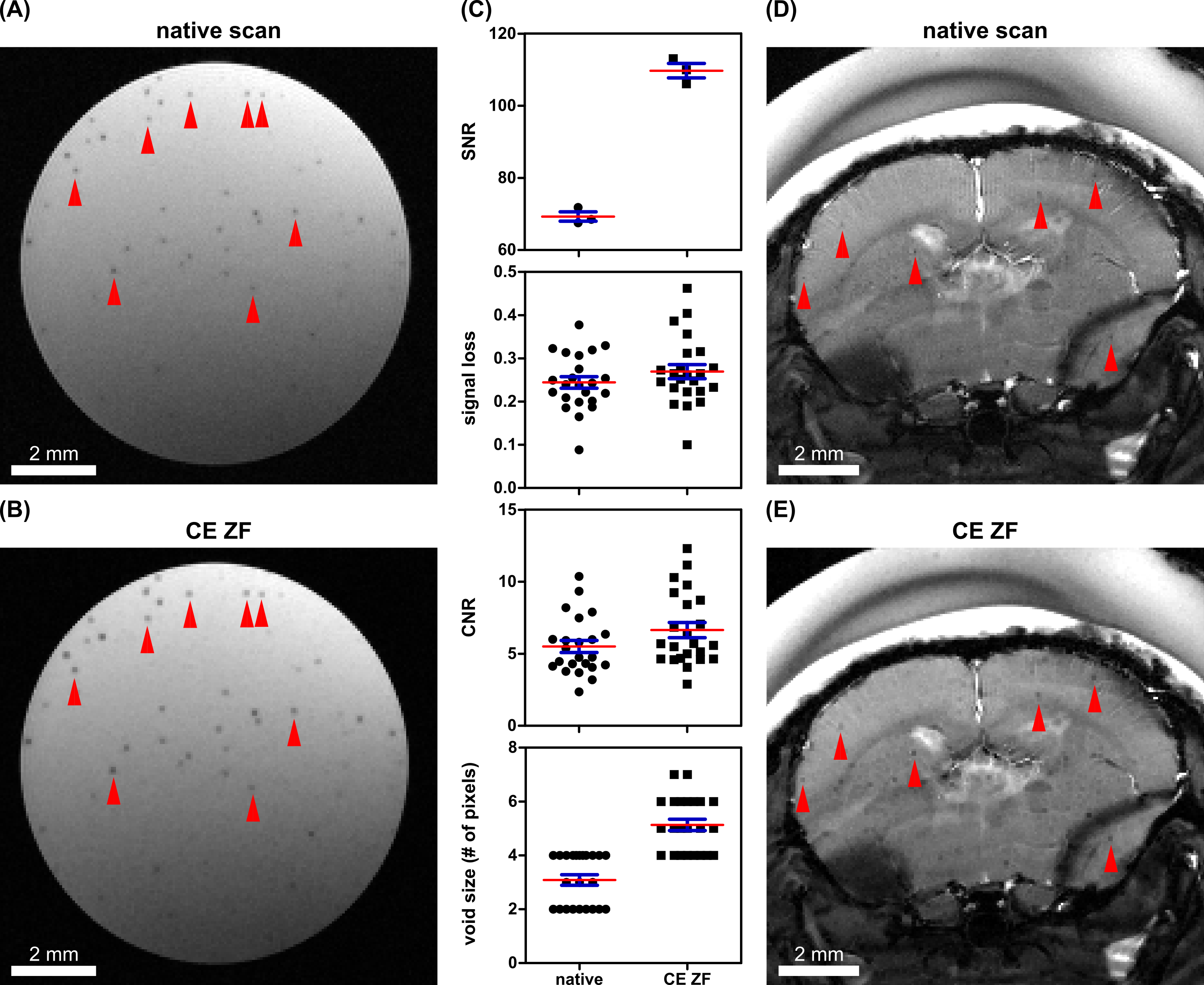


**Figure S1.** Retrospective contrast enhancement by zero-filling (CE ZF).
Exemplary images of phantom (A,B) and in vivo (D,E) measurements show that CE ZF (B,E) improved single particle/cell detection compared to the native scans (A,D). Red arrowheads indicate examples of single iron particles and iron-labeled cells, respectively. This observation was corroborated by quantification of image quality: In static phantoms, SNR increased from 69. 4 ± 1.1 in native scans to 109.9 ± 1.6 in images using CE ZF, signal loss from 0.24 ± 0.01 to 0.27 ± 0.02, CNR from 5.51 ± 0.41 to 6.65 ± 0.51, and void size from 3 ± 0 pixels to 5 ± 0 pixels. In vivo (n=3 measurements with n = 3 ROI each), the mean SNR improved from 20.8 ± 2.1 in native scans to 24.9 ± 3.2 in CE ZF images.
Individual dots in (C) represent individual ROIs for SNR calculation and individual hypointensities, i.e. iron particles, in signal loss, CNR and void size calculations in phantoms. Red bars indicate group means and the standard error of the mean (SEM) is shown in blue.


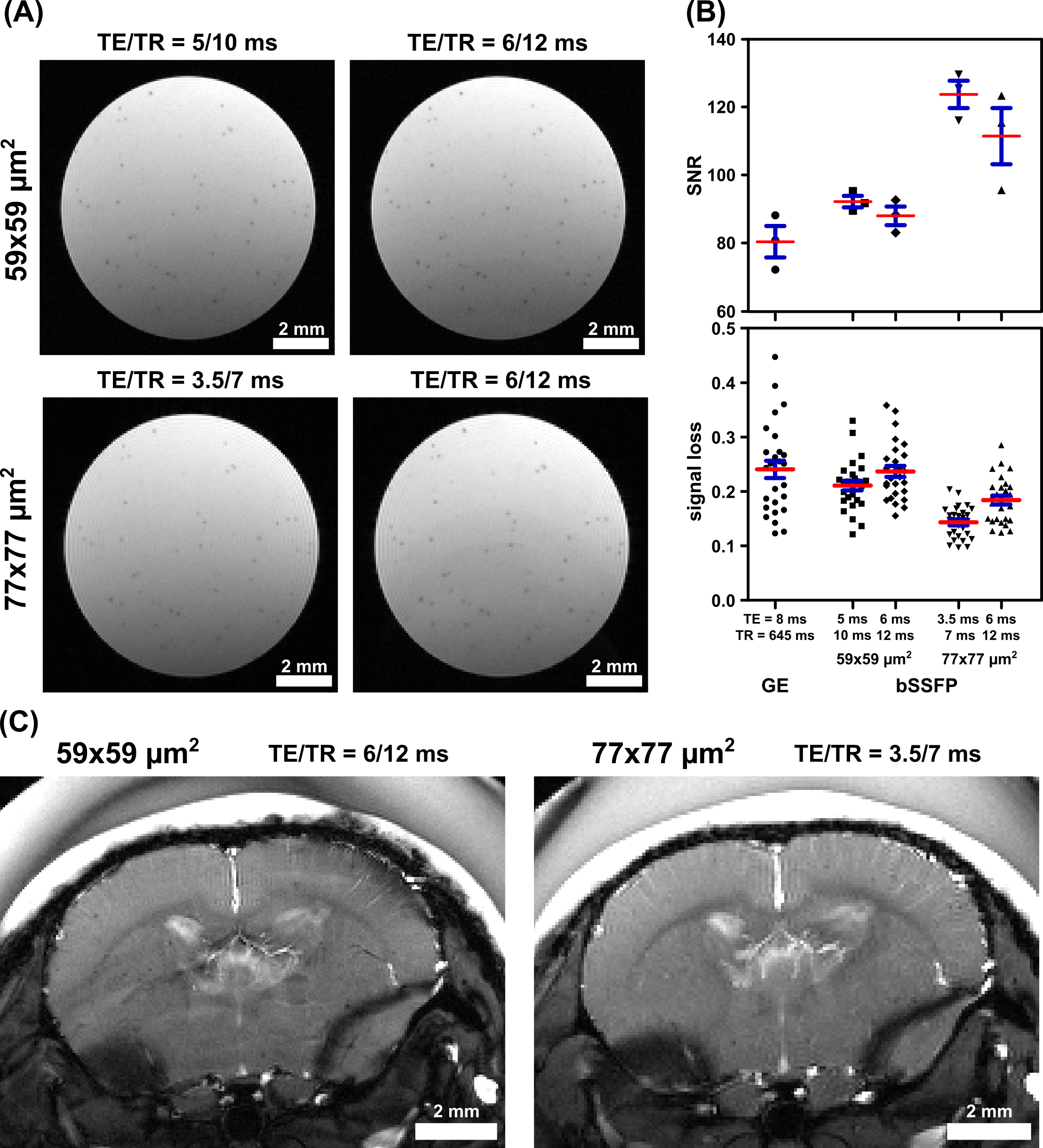


**Figure S2.** Effect of the echo time (TE) and repetition time (TR) on 2D Cartesian balanced steady-state free precession (bSSFP) imaging in time-lapse MRI.
The 2D Cartesian bSSFP in this study used a comparable long TR of 12 ms. While a shorter TR potentially reduces banding artifacts, the resulting shorter TE diminishes the effect of iron on signal loss hampering single cell detection.
In order to investigate this effect, first, phantom measurements were performed with a reduced TR of 10 ms (TE = 5 ms). Other imaging parameters were held constant as used in this study. A further reduction of TR at this spatial resolution of 59 x 59 µm^2^ was not possible due to gradient performances. Thus, spatial resolution was lowered to 77 x 77 µm^2^ and images were acquired for the shortest possible TR of 7 ms (TE = 3.5 ms). Image quality was assessed through SNR quantification and calculation of signal loss of individual particles. For comparison, bSSFP images with a TR of 12 ms as used in this study for both spatial resolutions as well as gradient echo images with the established time-lapse MRI sequence were acquired and analyzed.
(A) Exemplary images of phantom measurements using bSSFP with varying TE/TR and resolution suggested that contrast of signal voids caused by single iron particles is lower for a shorter TE/TR. Banding artifacts were not observed in any of the images.
(B) Quantification of SNR and signal loss confirmed this observation: Images acquired with a TR of 12 ms at high spatial resolution (59 x 59 µm^2^) generate a signal loss of 0.24 ± 0.01 comparable to the one achieved by GE (0.24 ± 0.02). Signal loss decreased to 0.21 ± 0.01 for the shorter TR of 5 ms. In low-resolution images (77 x 77 µm^2^), signal loss was 0.14 ± 0.01 and 0.18 ± 0.01 for a TR of 7 ms and 12 ms, respectively, thus smaller compared to high-resolution images. Again, a reduction of signal loss was observed when the shorter TE/TR was used. On the other hand, low-resolution images and short TE/TR yielded higher SNR, compared to high resolution and longer TE/TR as well as GE images (124 ± 4 and 111 ± 8 for TR of 7 ms and 12 ms at low spatial resolution, 92 ± 2 and 88 ± 3 for TR of 10 ms and 12 ms for high spatial resolution, 80 ± 5 for GE images).
For SNR quantification single dots represent individual regions-of-intereset (ROI) (n = 3), for signal loss individual hypointense spots, i.e. iron particles (n = 27). Bars indicate mean and standard error of the mean (SEM).
(C) Additionally, in vivo measurements revealed that banding artifacts occur for a long TR of 12 ms (TE = 6 ms) as well as for a shorter TR of 7 ms (TE = 3.5 ms). Here, examples of high-resolution images with a TR of 12 ms and low-resolution images with a reduced TR of 7 ms are shown.


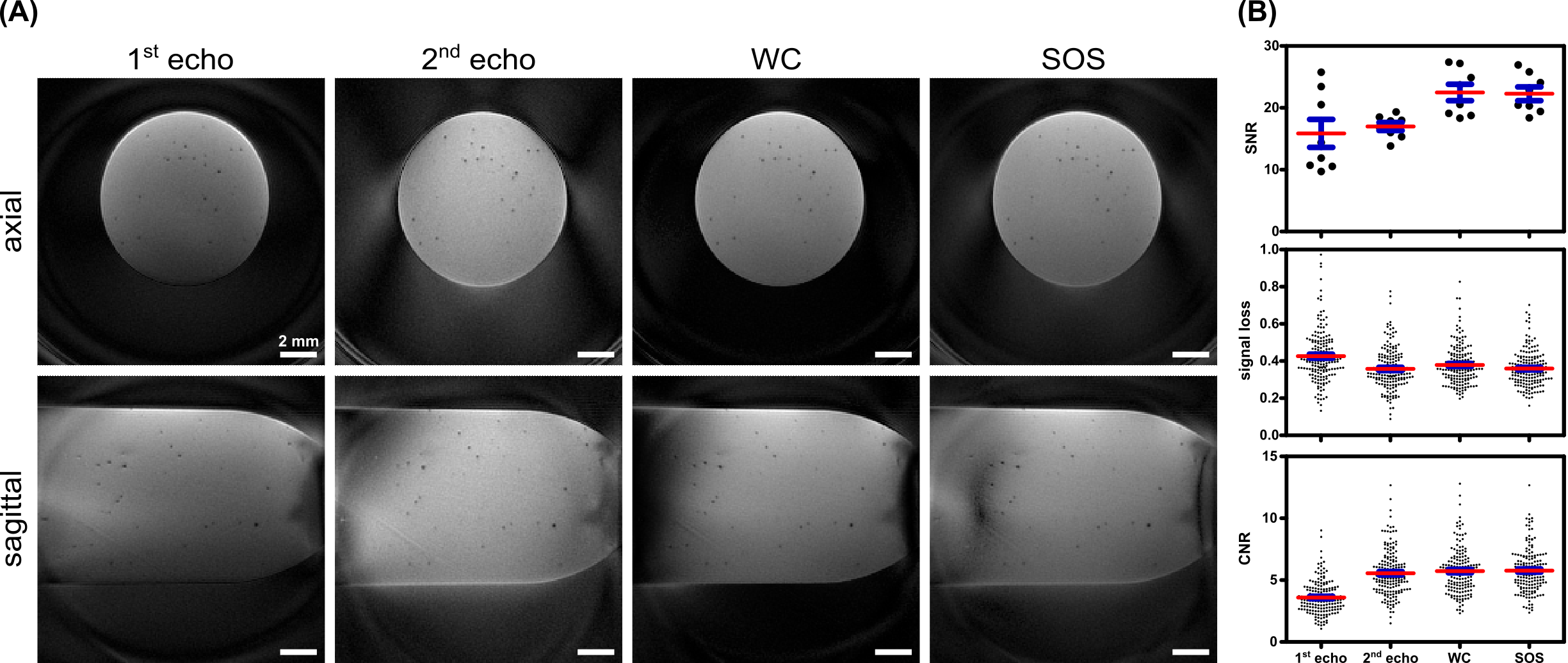


**Figure S3.** Comparison of first $I_{1}$ and second echo $I_{2}$, weighted (WC) ($I_{WC}=\left| \left| I_{1} \right|^{p}I_{1}+\left| I_{2} \right|^{p}I_{2} \right|^{\left( \frac{1}{1+p} \right)}; p=-0.5$) and sum-of-squares (SOS) combination ($I_{sos}=\left| \sqrt{{I_{1}}^{2}+{I_{2}}^{2}} \right|$). (A) Exemplary images of phantom measurements in axial (upper row) and sagittal view (lower row) are shown. (B) Image quality was compared measuring signal-to-noise ratios (SNR), as well as signal loss and contrast-to-noise ratios (CNR) of 175 single iron particles.

The first echo exhibits uneven signal distribution and low SNR (15.9 ± 2.3) and CNR (3.6 ± 0.1), yet the highest signal loss of single iron particles (0.43 ± 0.01). The second echo shows improved SNR (17.0 ± 0.6) and CNR (5.6 ± 0.1), with a slightly lower signal loss (0.36 ± 0.01) of individual particles. The SOS and WC methods yield comparable SNR (22.3 ± 1.1 and 22.5 ± 1.3, respectively), signal loss (0.36 ± 0.01 and 0.38 ± 0.01), and CNR (5.8 ± 0.1 and 5.7 ± 0.1), with SOS displaying dark regions within the phantom. The weighted combination minimizes dark regions to the field-of-view edges and achieves the highest SNR, signal loss, and CNR, making it the preferred method for 3D radial bSSFP echo combination.


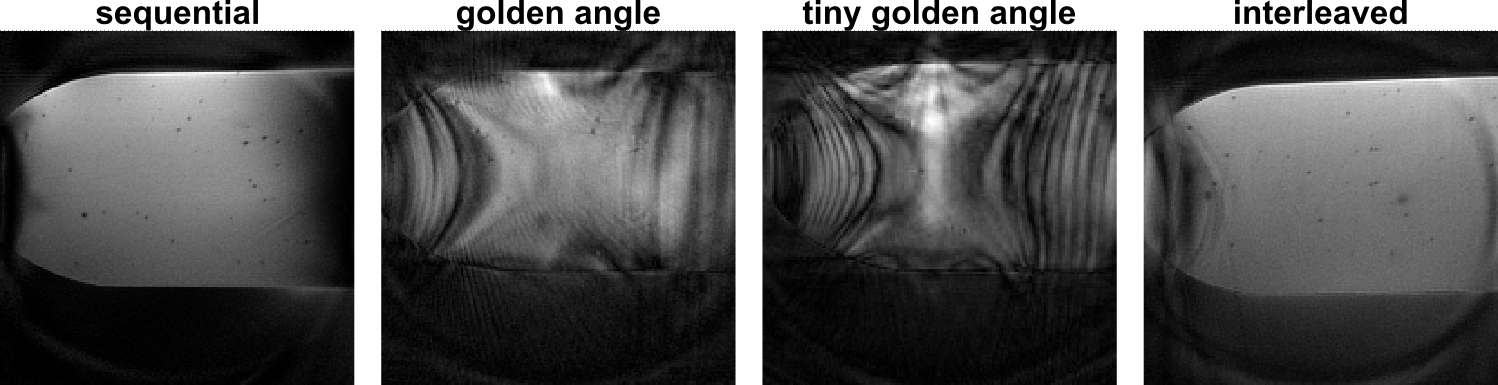


**Figure S4.** Eddy current artifacts in 3D radial balanced steady-state free precession (bSSFP) for different sampling schemes.
To enhance the temporal resolution of 3D radial bSSFP in time-lapse MRI, alternative sampling schemes were evaluated against standard sequential sampling. Here, representative sagittal slices of agar gel phantoms containing micron-sized iron particles are shown for sequential, golden angle, tiny golden angle (TINY-13), and interleaved (interleaved factor f=20) sampling. Imaging artifacts were observed as strong hypointense bands hindering single particle detection for (tiny) golden angle sampling.

**Video S1.** Rotating phantom measurements using 2D Cartesian balanced steady-state free precession (bSSFP) acquisition in time-lapse MRI. Single iron particles were identified as hypointense spots in the static (left) and rotating phantom (right). In the rotating case, 13 consecutive timeframes are shown. The rotational speed was 4.4 x 10^−3^ rpm with resulting particle speeds of up to 0.14 mm/min at the edge of the phantom. Slight temporal blurring of moving particles was observed as decreased signal loss and elongated shapes.

**Video S2.** Example of in vivo time-lapse MRI using 2D Cartesian balanced steady-state free precession (bSSFP) acquisition. Time-lapse MRI video of one slice with 20 timeframes is shown. An acquisition time of 1 min 42 s per timeframe resulted in a total scan time of 34 min. Single cells were resolved as hypointense spots and were followed dynamically (red arrowheads).

**Video S3.** Improvement of the temporal resolution in in vitro time-lapse MRI using an interleaved 3D radial acquisition scheme and CS reconstruction in balanced steady-state free precession (bSSFP) imaging.
Ten consecutive full timeframes and the corresponding 50 subframes of the accelerated reconstructions are shown. The rotational speed was 7.32 × 10^−3^ rpm. In the FS images (left), temporal blurring of moving particles was observed. In the US reconstruction (middle), motion distortion decreased, however noise increased. CS (right) improved image quality while keeping the high temporal resolution resulting in reduced temporal blurring compared to FS images.

**Video S4.** Example of in vivo time-lapse MRI using 3D radial balanced steady-state free precession (bSSFP) acquisition with interleaved ordering scheme. Time-lapse MRI video of an exemplary axial slice with 10 fully sampled (FS) timeframes and the corresponding 50 subframes of the accelerated reconstructions without (US) and with compressed sensing (CS) is shown. An acquisition time of 8 min per FS timeframe resulted in a total scan time of 80 min. Single cells were resolved as hypointense spots and were followed dynamically in FS and CS images (red arrowheads).
